# The involvement of purinergic signalling in obesity

**DOI:** 10.1007/s11302-018-9605-8

**Published:** 2018-04-04

**Authors:** Geoffrey Burnstock, Daniela Gentile

**Affiliations:** 1 Department of Pharmacology and Therapeutics, The University of Melbourne, Parkville, Melbourne, Victoria 3010 Australia; 2 The Florey Institute of Neuroscience and Mental Health, Parkville, Melbourne, Australia; 3 Autonomic Neuroscience Centre, University College Medical School, Rowland Hill Street, London, NW3 2PF UK; 4 Department of Clinical and Experimental Medicine, University of Pisa, Via Roma 55, 56126 Pisa, Italy

**Keywords:** Obesity, Brown and white adipocytes, Hypothalamus, ATP, Adenosine, Purinergic receptors

## Abstract

Obesity is a growing worldwide health problem, with an alarming increasing prevalence in developed countries, caused by a dysregulation of energy balance. Currently, no wholly successful pharmacological treatments are available for obesity and related adverse consequences. In recent years, hints obtained from several experimental animal models support the notion that purinergic signalling, acting through ATP-gated ion channels (P2X), G protein-coupled receptors (P2Y) and adenosine receptors (P1), is involved in obesity, both at peripheral and central levels. This review has drawn together, for the first time, the evidence for a promising, much needed novel therapeutic purinergic signalling approach for the treatment of obesity with a ‘proof of concept’ that hopefully could lead to further investigations and clinical trials for the management of obesity.

## Introduction

Obesity, defined as abnormal or excessive fat accumulation, represents a major health issue, with an alarmingly increasing prevalence in developed countries, caused by the dysregulation of energy balance. The World Health Organization in 2016 reported that more than 1.9 billion adults aged 18 years and older were overweight [body mass index (BMI) > 25 kg/m^2^], and of these, over 650 million adults were obese (BMI > 30 kg/m^2^) (http://www.who.int/mediacentre/factsheets/fs311/en/). The imbalance of energy underlying obesity is due to several factors, including genetic predisposition, individual metabolism, excessive caloric and food intake and insufficient physical activity, leading to an increase in adipose tissue. In recent years, the crucial role of adipose tissue in the regulation of energy metabolism has been recognised, which not only dynamically accumulates and releases lipids but also acts as an endocrine organ [1]. Indeed, adipose tissue produces a variety of humoral factors known as adipocytokines (i.e. leptin, adiponectin, resistin and visfatin) that contribute to the regulation of appetite and satiety, fat distribution, insulin secretion and sensitivity, energy expenditure, endothelial function, inflammation and blood pressure [2, 3].

In mammals, adipose tissue can be divided into brown and white adipose tissues [2]. White adipose tissue represents the vast majority of adipose tissue in the organism and is the site of energy storage, whereas brown adipose tissue burns energy for thermogenesis [2]. Adipocytes are the main components of adipose tissue, and adipogenesis has two distinct phases: early differentiation of the adipocytes from a multipotent stem cell and terminal differentiation of preadipocytes into mature adipocytes [4]. Epidemiologic studies have suggested that the number of adipocytes in an adult are approximately constant whether they are lean or obese [5]. Moreover, significant weight gain or loss in adults is not accompanied by respective increases or decreases in the number of adipocytes, rather adipocyte size is correlated with adult adiposity. These observations support the notion that the number of adipocytes a person will have is determined during childhood and adolescence. Indeed, in line with this evidence, environmental exposure in early life can influence adipocyte number and has the potential to greatly increase the total body fat mass and may contribute to the development of obesity in adults [5].

Regulation of energy homeostasis is highly controlled by the central nervous system (CNS). Indeed, it receives and integrates signals conveying energy status from the periphery, such as leptin and insulin, leading to modulation of food intake [6]. The autonomic nervous system (ANS) plays an important role in the response to such signals, innervating peripheral metabolic tissues, including brown and white adipose tissues [7]. The ANS consists of two parts: the sympathetic and parasympathetic nervous systems. Since the ANS is involved in the regulation of the cardiovascular system, hormonal secretion and energy balance, it is plausible that altered regulation of either the parasympathetic or sympathetic branches, or both, may contribute to the development of obesity and related metabolic comorbidities [8]. Depression of sympathetic and parasympathetic activity has been associated with increasing body fat, but whether this is causal or consequential was not resolved. Moreover, sympathetic denervation has been reported to lead to an increase in white adipocyte cell number and fat pad mass [9].

Currently, therapeutic strategies against obesity have been largely ineffective, such as 5-hydroxytryptamine modulators, β_3_ adrenoceptor agonists, lipase inhibitors, melanocortin 4 inhibitors, leptin agonists and ghrelin antagonists [10]. The development of novel anti-obesity drugs based on our current understanding of energy homeostasis is required. The present review explores the possible involvement of purinergic signalling in obesity.

Purinergic signalling [i.e. adenosine 5′-triphosphate (ATP) acting as an extracellular signalling molecule] was proposed in 1972 (see [11]). After early resistance to the concept, when receptors for ATP and adenosine were cloned and characterised in the early 1990s, it was generally accepted and there has been an explosion of interest in the physiology and pathophysiology of purinergic signalling (see [12]). Selective agonists and antagonists to both adenosine (P1) receptor subtypes (A_1_, A_2A_, A_2B_, A_3_) and P2X ion channel receptor subtypes for ATP (P2X_1–7_) and P2Y G protein-coupled receptor subtypes to ATP, adenosine 5′-diphosphate (ADP), uridine 5′-triphosphate (UTP) and uridine 5′-diphosphate (UDP) (P2Y_1_, P2Y_2_, P2Y_4_, P2Y_6_, P2Y_11–14_) have been developed and clinical trials initiated that have led to the use of purinergic agents for the treatment of several diseases, including clopidogrel, a P2Y_12_ receptor antagonist for the treatment of stroke and thrombosis, a P2Y_2_ long-term agonist for the treatment of dry eye and adenosine A_1_ agonists for the treatment of tachycardia. Clinical trials are currently in progress to explore purinergic agents for the treatment of osteoporosis, chronic cough, visceral pain, bladder incontinence, cancer and neurodegenerative diseases (see [13]).

## Purinergic control of brown adipocytes

Brown adipocytes, located in specific areas of the body, express constitutively high levels of thermogenic genes making them specialised in energy expenditure and therefore a potential target for anti-obesity therapies [14]. There are also beige cells, which are inducible ‘brown-like’ adipocytes that develop in white fat in response to various activators. The activities of brown and beige fat cells reduced obesity in mice, an effect similar to that seen in lean humans [14], in addition to causing antidiabetic effects [15]. Lipid synthesis by brown adipocytes in rats was increased by sympathetic nerve stimulation, and it was recognised that this was not solely attributable to the action of noradrenaline but included some non-adrenergic mechanisms [16]. The thermogenic function and growth of brown tissue is also controlled by the sympathetic nervous system in rats, but antidromic activity by sensory nerves may also be involved [17]. High-fat diet (HFD) in rats has been associated with a reduction in sympathetic activity to brown adipose tissue [18].

ATP, released as a cotransmitter from sympathetic nerves, was reported to elicit substantial increases in total membrane capacitance of rat brown fat cells, probably via P2Y receptors [19]. ATP mobilises Ca^2+^ from intracellular stores, supporting the view that P2Y receptors were involved [20]. ATP was also shown by these authors to exert a potent inhibitory effect on the influx of Ca^2+^ in cultured adult brown adipocytes [20]. Evidence was presented to suggest that modulation of voltage-gated potassium currents in rat brown adipocytes by ATP might be important in controlling adipocyte growth and development [21]. Ca^2+^-ATPase (SERCA), a family of membrane-bound ATPases that are able to translocate Ca^2+^ ions across the membrane using the chemical energy derived from ATP hydrolysis, was shown to generate heat in the presence of Ca^2+^ concentrations similar to those occurring during adrenergic stimulation in rat brown adipocyte mitochondria [22].

Multiple P2 receptor subtype mRNA was later identified in rat brown fat cells: P2Y_2_, P2Y_6_ and P2Y_12_ metabotropic receptors and P2X_1_, P2X_2_, P2X_3_, P2X_4_, P2X_5_ and P2X_7_ receptors; ATP, ADP, UTP and UDP increased intracellular Ca^2+^ [23].

Adenosine is present in adipose tissue after breakdown by ectoenzymes of ATP released as a cotransmitter from sympathetic nerves and from adipocytes. Adenosine was shown to regulate hamster brown adipose tissue respiration at an early metabolic step of the stimulus-thermogenesis sequence [24]. Adenosine increased lipolysis and induced thermogenesis in brown adipocytes via A_2A_ receptors, and A_2A_ agonists were shown to counteract HFD-induced obesity in mice [25].

## Purinergic control of white adipocytes

White adipocytes are the major energy reservoir in mammals, and they play a crucial role in the maintenance of energy homeostasis [26].

ATP increased cell membrane capacitance in rat white adipocytes, similar to that produced in brown adipocytes, indicating that the electrophysiology of both kinds of adipocytes is very similar in their response to ATP [27].

Rat white adipocytes express at least two P2Y receptor subtypes, and activation of P2Y_11_ receptors may mediate inhibition of leptin production and stimulation of lipolysis, suggesting an important role of purinergic transmission in white adipocyte physiology [28]. A combination of ATP and Ca^2+^ has been reported to augment human white adipocyte vesicular release of adiponectin [29]. This study also investigated the cellular mechanisms involved in the regulation of human white adipocyte exocytosis/secretion by monitoring the membrane capacitance. The authors showed that protein kinase A-independent mechanisms could be correlated with a release of adiponectin vesicles, elucidating previously unknown cellular mechanisms involved in the regulation of white adipocyte exocytosis/secretion [29]. Disturbance of adiponectin secretion in individuals with obesity highlights the control of adipokinase release by ATP. Reduction in the plasma level of adiponectin in subjects with obesity precedes the reduction in insulin sensitivity and onset of diabetes [30].

Adenosine monophosphate (AMP) kinase, a cellular energy sensor activated by cellular stresses and also by leptin and adiponectin, has fat-reducing effects in mammalian white adipose tissue and is a potential target for obesity treatment [31]. The authors suggested that chronic AMP kinase activation acts by remodelling adipocyte glucose and lipid metabolism, which then enhances the ability of adipose tissue to remove energy and reduce adiposity [31].

In isolated rat white adipocytes, adenosine, produced following breakdown of ATP, acts as a positive regulator for insulin in the release of leptin via an activation of A_1_ receptors that involves the phospholipase C-protein kinase C pathway [32].

## Hypothalamic purinergic nervous control of obesity

In the last decade, many studies have highlighted a fundamental role of the CNS, in particular the arcuate nucleus of the hypothalamus (ARH), in the regulation of food intake and energy balance in mammals [33]. In mammals, the ARH is accessible to circulating signals of energy balance, via the underlying median eminence, as this region of the brain is not protected by the blood-brain barrier [34]. They showed, in particular, that the ARH integrates neurohormonal signalling from the gut and adipose tissue, communicating nutrient availability, including ghrelin, insulin, glucose, leptin and UDP. The ARH contains two primary neuron populations that integrate signals of nutritional status and influence energy homeostasis [35]. One neuronal circuit inhibits food intake, via α-melanocyte-stimulating hormone, and cocaine- and amphetamine-regulated transcripts [36]. The other neuronal circuit stimulates food intake, via the expression of neuropeptide Y and agouti-related peptide (AgRP) [37]. Several studies aimed at finding novel approaches for the management of obesity have focused on the critical role of AgRP neurons in the regulation of appetite, reporting that their direct activation rapidly increases food intake. In contrast, AgRP neuron inhibition [38] or ablation dramatically decreases feeding [39] in mice.

**Fig. 1:**
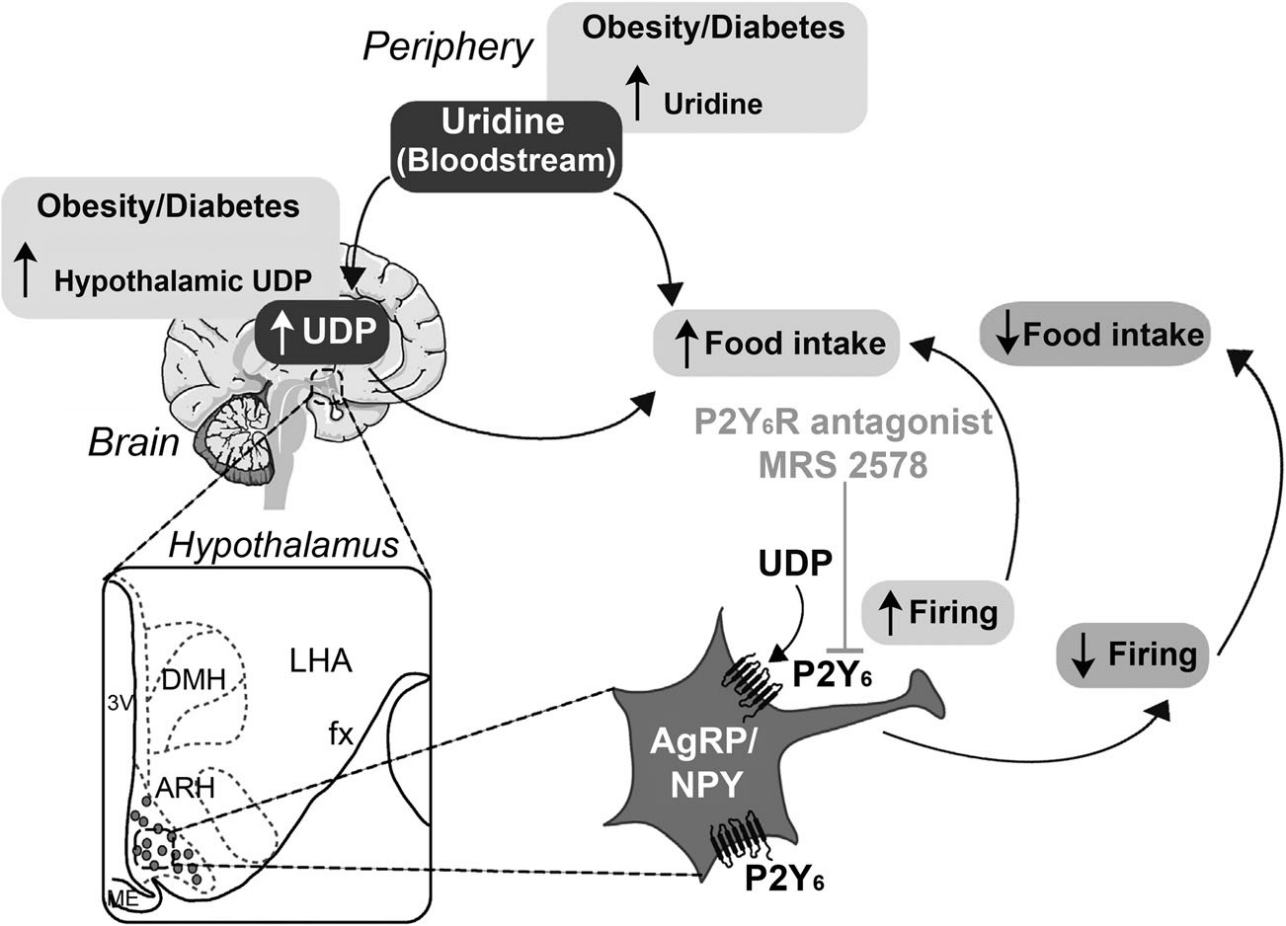
The UDP-selective P2Y_6_ receptor controls orexigenic AgRP neurons and food intake regulation. The central nervous system (CNS), in particular the arcuate nucleus of the hypothalamus (ARH), integrates neurohormonal signalling from the periphery, such as uridine 5′-diphosphate (UDP). The P2Y_6_ receptor, a member of the P2Y G protein-coupled receptor family, is highly expressed in the ARH, particularly in AgRP neurons. The P2Y_6_ receptor is selective for the nucleotide UDP, whose synthesis in the CNS depends on the salvage pathway, which is directly controlled by the peripheral supply of the precursor metabolite uridine, typically increased in obesity/diabetes. Pharmacological blocking of P2Y_6_ receptor activation in the CNS, with the selective P2Y_6_ receptor antagonist MRS 2578, inhibits feeding in mice (modified from [41] and reproduced with permission from Elsevier)

Of the regulators of the central control of feeding behaviour, the family of G protein-coupled receptors has significant therapeutic potential, due to their involvement in the regulation of physiological responses to hormones, neurotransmitters and environmental stimulants [40]. It was shown in mice that the UDP-selective P2Y_6_ receptor, a P2Y G protein-coupled receptor, is highly expressed in the ARH, particularly in AgRP neurons [41]. The authors provided the first evidence that the activation of P2Y_6_ receptor signalling, by UDP, increases firing rate and feeding in lean mice [41]. Pharmacological blocking of P2Y_6_ receptor activation in the CNS, with the selective P2Y_6_ receptor antagonist MRS 2578, inhibits feeding in mice. These authors showed in a more recent study that the ability of centrally applied UDP to acutely promote feeding is retained in diet-induced obese mice [42]. In contrast, pharmacological blocking of P2Y_6_ receptor activation in the CNS via intracerebroventricular application of MRS 2578 inhibits food intake in obese mice (see Fig. 1). Moreover, both conventional and AgRP-restricted P2Y_6_-deficient animals exhibit reduced obesity as well as improved whole-body insulin sensitivity when exposed to long-term HFD feeding. Thus, although further investigations are needed, P2Y_6_ receptors could represent a potential therapeutic target for the prevention and treatment of obesity and related insulin resistance. Furthermore, since P2Y_6_ receptors are also expressed on activated microglia in the hippocampus of rats [43] and considering that obesity promotes hypothalamic inflammation, including the activation of microglia [44], this purinergic receptor could hold a potential pathophysiological role in inflammatory processes within the CNS induced by HFD.

## Regulatory roles of ATP and P2 receptors in obesity

**Fig. 2:**
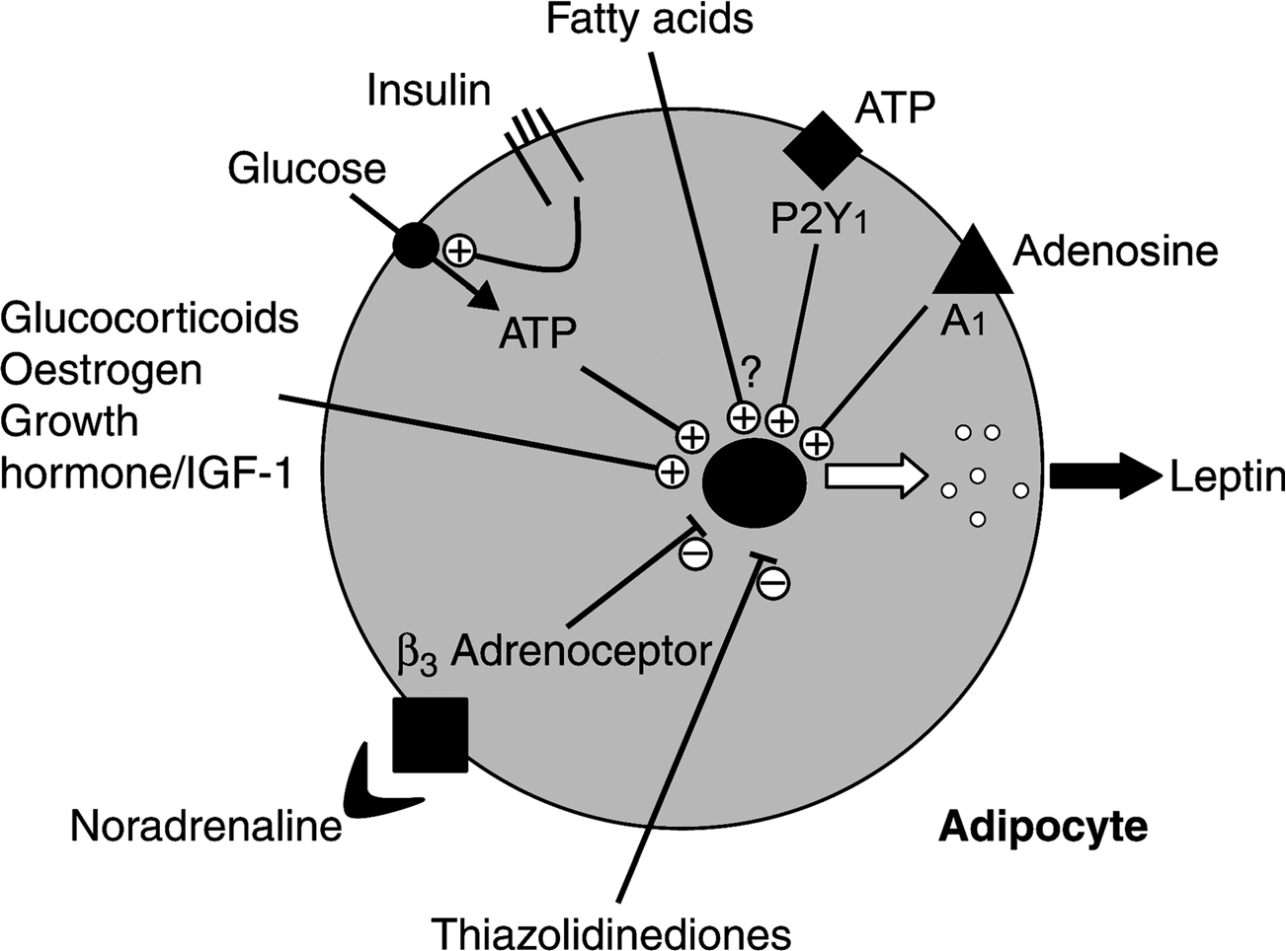
Factors influencing leptin synthesis and secretion. Insulin-mediated glucose uptake determines the rate of glucose metabolism in adipose tissue, and the subsequent generation of ATP is a major stimulus for leptin production and secretion. Some fatty acids may also have an effect (indicated with question mark). Hormones such as glucocorticoids, oestrogen and growth hormone also stimulate leptin secretion. Catecholamines, via the β_3_ adrenoceptor, tend to inhibit leptin production. The antidiabetic thiazolidinedione drugs also inhibit leptin production, but the mechanism is not known. ATP acts on P2Y_1_ receptors to mediate regulation of leptin secretion from adipocytes in lean, but not obese mice, and adenosine acts on A_1_ receptors to increase leptin secretion (modified from [46] and reproduced with permission from Elsevier)

In a review entitled ‘Leptin and the control of obesity’, it was stated that ‘ATP is a major stimulus for leptin production and secretion’ [10, 45, 46] (see Fig. 2). Bullock and Daly reviewed the evidence for sympathetic nerve innervation of perivascular adipocytes and the function of ATP, released as a sympathetic cotransmitter with noradrenaline, which inhibits lipolysis [47]. In addition, there is strong evidence that the vagus nerve is involved in the development of diet-induced obesity (see [48]) and, since ATP is also a cotransmitter with acetylcholine in vagal nerves, it may be involved in its mechanisms. The likely source of the ATP is sympathetic nerves, and ATP was reported to inhibit insulin-stimulated glucose transport and glycogen synthase in rat fat cells [49].

In a later study also performed on rats, ATP was shown to have a strong effect, while adenosine a mild inhibitory effect, on insulin-stimulated glucose transport [50]. It is possible that ATP, released as a cotransmitter from sympathetic nerves, mediates the long-term effects of leptin on blood pressure involved in obesity hypertension [51]. ATP has antihyperlipidaemic activity by decreasing serum triglyceride levels in rabbits fed a HFD and in hyperlipidaemic patients, suggesting that ATP supplementation could provide an effective approach to control triglyceride levels in obesity [52]. A later paper provided evidence that ATP stimulated lipogenesis in rat adipocytes via a P2 receptor (probably a P2X receptor), defining a signalling system involved in the regulation of fat stores in these cells independent from established hormones [53]. ATP increased the membrane area, which was correlated with the increase in membrane current in single rat adipocytes [54]. High concentrations of ATP have been reported to induce inflammatory responses and insulin resistance generation in rat adipocytes [55]. In line with this concept, reduced hepatic ATP stores have been found to be more common in overweight and obese than in lean subjects [56].

### P2X receptors

Of the P2X receptors, an involvement of both P2X_2_ and P2X_7_ receptors has been identified. Obesity promoted a decrease in the expression of P2X_2_ receptors on enteric neurons of obese male mice [57]. Human adipocytes from metabolic patients express functionally active P2X_7_ receptors, which modulate the release of inflammatory molecules such as interleukin-6, tumour necrosis factor-α and plasminogen activator inhibitor-1, in part via inflammasome activation [58]. Moreover, these cells also exhibited enhanced P2X_7_ receptor expression, which might contribute to the subclinical inflammatory status characterising these patients and conferring on them an increased cardiovascular risk [58]. In addition, Rossi and colleagues, in line with this evidence, demonstrated that patients affected by metabolic syndrome showed an enhancement of P2X_7_ receptor expression and inflammasome activation compared to control patients [59]. However, it was claimed in another study on mice that ATP activation of P2X_7_ receptors was not involved in inflammasome activation in adipose tissue [60]. The P2X_7_ receptor has been reported to be the primary mediator of oxidative stress-induced exacerbation of inflammatory liver injury in obese mice [61]. Moreover, the fact that P2X_7_ receptor antagonists significantly decreased carbon tetrachloride exacerbation of liver injury in obesity paved the way for future investigations using the antagonists as potential therapeutic molecules in treating steatohepatitis in obesity in its early phase [61]. Of note, in metabolically unhealthy obese subjects, stromal vascular cells showed upregulation of P2X_7_ receptors, which are involved in the chronic inflammation of visceral adipose tissue underlying the metabolic changes in obesity [62]. In P2X_7_ knockout mice, there is abnormal fat distribution, suggesting that P2X_7_ receptors mediate regulation of adipogenesis and lipid metabolism in age- and sex-dependent manners [63]. ATP-induced inflammation, via P2X_7_ receptors, drives tissue-resident Th17 cells in metabolically unhealthy obese subjects, and it was suggested that the manipulation of purinergic signalling might represent a new therapeutic target to shift the CD4^+^ T cell balance under inflammatory conditions [64]. Sulphur-containing AMP and guanosine monophosphate analogues can be hydrolysed to hydrogen sulphide by rat perivascular adipose tissue when P2X_7_ receptors are activated [65].

### P2Y receptors

ATP, acting via P2Y receptors, enhanced the migration of preadipocytes and increased adipocyte differentiation in a mouse cell line [66]. P2Y_1_ receptors mediate regulation of leptin secretion from adipocytes in lean, but not in obese mice [67]. ATP, acting via P2Y_1_ receptors, contributes to the cell surface F1F0-ATP synthase-mediated intracellular triacylglycerol accumulation in mouse adipocytes [68]. Mouse P2Y_4_ receptors are negative regulators of cardiac adipose-derived stem cell differentiation and cardiac fat formation [69]. Therefore, these receptors could be a potential therapeutic target in the regulation of the cardioprotective function of cardiac fat. Myenteric neurons from P2Y_13_ receptor knockout mice or treatment with P2Y_13_ receptor antagonists are resistant to HFD- and palmitic acid-induced neuronal loss; consequently, P2Y_13_ receptor antagonism might constitute a novel therapeutic strategy in patients affected by intestinal dysmotility involving neuropathy [70]. P2Y_6_ receptor agonists enhance glucose uptake in mouse adipocytes and skeletal muscle cells [71]. As previously described, the activation of P2Y_6_ receptor signalling, by UDP, increases firing rate and feeding in lean mice [41].

### ATP-sensitive K^+^ channels

Extracellular ATP modulates several ionic channels, such as K^+^ channels. Modulation of ATP-sensitive K^+^ (K_ATP_) channel activity has been the basis of numerous pharmacological studies since these channels are abundant in a variety of tissues and species. K_ATP_ channel activity is coupled with insulin resistance in obesity and type 2 diabetes in mammals [72]. Indeed, insulin activates K_ATP_ channels in hypothalamic neurons of lean but not obese rats, suggesting that hypothalamic K_ATP_ channels have a crucial role in the physiological regulation of food intake and body weight [73]. K_ATP_ function was decreased in obese rats, along with impaired vasodilation in response to exercise [74]. This evidence suggested that the decreased sensitivity of K_ATP_ channels could potentially limit muscle blood flow during exercise, a treatment option known to improve glucose, lipid and weight control [74]. Evidence suggested that K_ATP_ channel-deficient mice exhibit hyperphagia but are resistant to the induction of obesity by a HFD [75].

### ATP-binding cassette transporters

ATP-binding cassette (ABC) transporters (ABCA1, ABCG1, ABCG5 and ABCG8) are examples of ATP-dependent pumps involved in mediating macrophage cholesterol efflux in animal models and in vitro experiments [76, 77]. The ABC transporter A1 R230C variant was reported to affect high-density lipoprotein cholesterol levels and to be associated with obesity and obesity-related comorbidities in the Mexican population [78]. The ABC transporter G8 gene was shown to be a determinant of apolipoprotein B-100 kinetics in a study of Australian overweight/obese men [76]. The R219K polymorphism of ABC transporter A1 is related to low high-density lipoprotein level in overweight/obese Thai males [79]. The expression of ABC transporter A1 in monocytes was reduced in Chinese overweight and obese patients, and this was associated with the impairment of cholesterol efflux from monocyte-derived macrophages [80]. Since adipocyte ABC transporter G1 promoted triglyceride storage and fat mass growth, it might represent a potential therapeutic target in the control of fat accumulation [81].

### Other purinergic therapeutic possibilities

Cell surface H^+^-ATP synthase has been claimed to be a potential molecular target for anti-obesity drugs. Of note, treatment with small molecule inhibitors of H^+^-ATP synthase or antibodies against H^+^-ATP synthase subunits leads to a decrease in cytosolic lipid droplet accumulation in differentiated adipocytes [82].

Transcriptional regulation of the gene for ATP citrate lyase (one of the lipogenic enzymes) by glucose/insulin and leptin was investigated in hepatocytes and adipocytes of normal and genetically obese rats. In the presence of glucose/insulin, the chloramphenicol acetyltransferase activities were markedly increased in hepatocytes of lean rats but were not significantly increased in those of obese rats [83].

It has been suggested that animal and human obesity is associated with reduction of tissue Na^+^/K^+^-ATPase, linked to hyperinsulinemia, influencing thermogenesis and energy balance [84].

Typical signs of Cushing’s syndrome and side effects of prolonged glucocorticoid treatment are features of the metabolic syndrome, such as central obesity with insulin resistance and dyslipidaemia. Changes in AMP-activated protein kinase have been proposed as a novel mechanism to explain the deposition of visceral adipose tissue and the consequent central obesity in individuals with Cushing’s syndrome [85].

## Regulatory role of adenosine and P1 receptors in obesity

In early studies on rat adipocytes, adenosine was shown to inhibit lipolysis elicited by noradrenaline [86] due to its inhibition of adenylate cyclase and cyclic AMP production by a guanosine triphosphate-dependent process [87]. Adenosine was shown to be rapidly taken up by isolated fat cells and incorporated into ATP, which, after release, was broken down by ectoenzymes to adenosine [88]. Theophylline, an adenosine antagonist, and dipyridamole, an inhibitor of adenosine uptake, were shown to enhance lipolysis [89]. Sites on adipocyte membranes that bind [^3^H]adenosine were demonstrated and identified as adenosine (P1) receptors [90]. Three subtypes of P1 receptors were described on adipocytes obtained from epididymal and perirenal fat pads [91]. A_1_ receptors were shown to be present on human adipocytes [92], and in rats, white adipocytes were more responsive than brown adipocytes to inhibition of lipolysis by activation of A_1_ receptors [93]. Cloning, expression and characterisation of the A_1_ receptor on mouse and human adipose tissues were reported [94]. A_1_ receptor activation results in an increase of adipocyte leptin secretion in rats [95]. A_1_ receptors are highly expressed in adipose tissue, and their contribution to the regulation of lipolysis in pathological conditions like insulin resistance, diabetes and dyslipidemia, where free fatty acids play an important role, has been examined [96]. Agonists to A_1_ receptors are in clinical trials for obesity. The A_2_ adenosine receptor subtype, which is positively coupled to adenylate cyclase, was shown to be expressed by preadipocytes, but not activated adipocytes, suggesting that adenosine might play as a bimodal regulatory signal in adipose tissue development in rats [97]. There were contrasting effects of transfected human A_1_ and A_2B_ receptors into a murine osteoblast precursor cell line, 7F2. A_1_ receptors mediated adipocyte differentiation, whereas A_2B_ receptors mediated inhibition of adipogenesis and stimulated an osteoblastic phenotype [98]. Activated transfected human A_1_ receptors initiated differentiation of mouse preadipocyte cells [99]. Deletion of adenosine A_1_ receptors in knockout mice should increase lipolysis and decrease lipogenesis, but an increased fat mass was observed, indicating that there are other actions mediated by A_1_ receptors [100]. Differentiation of rat mesenchymal stem cells to adipocytes was accompanied by significant increases in the expression of A_1_ and A_2A_, and their activation was associated with increased adipogenesis [101]. There is impaired glucose tolerance in A_1_ receptor knockout mice [102].

Insulin, as well as adenosine, is antilipolytic in rats [103]. Also in rats, adenosine modulation of the stimulation of glucose metabolism in adipocytes by insulin was shown to be mediated by different mechanisms from that mediated by oxytocin [104]. Adenosine, via A_1_ receptors, increased insulin sensitivity and inhibited lipolysis in adipocytes. After prolonged incubation of rat adipocytes with an A_1_ adenosine receptor agonist, *N*^6^-phenylisopropyl adenosine, there was downregulation of the receptor and insulin resistance [105]. Over-expression of A_1_ receptors in adipose tissue protects mice from obesity-related insulin resistance, and it was suggested that A_1_ receptor activation should be considered as a potential therapeutic target for the treatment of obesity-related insulin resistance and type 2 diabetes [106]. Insulin resistance in obese Zucker rats is tissue specific, and BWA1433, an adenosine receptor antagonist, improved glucose tolerance by increasing glucose uptake in skeletal muscle, while decreasing glucose uptake by adipose tissue [107]. There was enhanced sensitivity to both lipolytic stimuli and adenosine suppression of lipolysis in isolated fat cells from streptozotocin-diabetic rats [108].

Adipocytes from hypothyroid rats respond to adenosine, but not to adrenaline, with increased glycerol release [109]. Short-term hyperthyroidism modulates adenosine receptors and adenylate cyclase in rat adipocytes [110]. Studies of membranes from hyperthyroid rats showed no significant alteration on the expression of A_1_ receptors [111]. Adenosine increases blood flow and glucose uptake in adipose tissue of dogs [112] and in brown adipose tissue of rats [113].

In brown subcutaneous abdominal fat cells from subjects with obesity, the antilipolytic effect of an adenosine analogue was markedly attenuated as compared to that in fat cells from normal-weight subjects [114]. A reduction in the P1 receptor number in adipocyte plasma membranes and reduced adenosine sensitivity in human obesity were reported [115]. Inhibition of isoprenaline-stimulated lipolysis by an adenosine receptor agonist was much attenuated in cells from patients that were massively obese, compared to normal-weight control subjects [116]. Obese rats show reduced adenosinergic modulation of ventilatory responses to acute and sustained hypoxia [117]. It was concluded that this was due to depressed peripheral excitatory mechanisms and to enhanced adenosinergic central depression mechanisms. An adenosine deaminase polymorphism was shown to be associated with obesity, and adenosine receptor agonists were recommended as therapeutic targets for obesity and dyslipidemia [118]. Data was presented to suggest that inhibition of lipolysis by adenosine appears to be greater in African-American women with obesity and that this might possibly be one explanation for the observation that African-American women with obesity have more difficulty in losing weight than Caucasian women with obesity [118].

A HFD induced changes in glucose homeostasis, inflammation and obesity. A_2B_ receptors were upregulated in lean mice by a HFD, while A_2B_ receptor knockout mice under this diet developed greater obesity and signs of type 2 diabetes [119]. The authors showed further that in human subjects with obesity, A_2B_ receptor expression correlated strongly with expression of the insulin receptor substrate 2, and suggested that A_2B_ receptor agonists have potential for the treatment of type 2 diabetes and obesity. A recent study by Antonioli and coworkers reported that A_2B_ receptors participate to obesity-related enteric dysmotility, modulating the activity of excitatory tachykininergic nerves in HFD mice [120]. A review about adenosine and adipogenesis is available [121]. High plasma levels of adenosine were found in children with obesity [122] and in overweight pregnant women [123]. Non-alcoholic fatty liver disease is an obesity-related condition. A study provided insight into the lipolytic actions of caffeine (a P1 receptor antagonist) through autophagy in mammalian liver and its potential beneficial effects in non-alcoholic fatty liver disease [124]. Obesity causes macrophage activation, which, in turn, causes insulin resistance in target organs. Adenosine, acting via A_2B_ receptors, prevented adipose tissue inflammation and insulin resistance; therefore, it suggests a possible therapeutic strategy for inhibiting adipose tissue inflammation [125]. Evidence was presented that there may be a role for the ectonucleotidase CD73 and A_2A_ receptors in inflammation observed in patients with type 2 diabetes and obesity mediated via apoptosis [126]. Adenosine protects rats from a HFD by reducing glucose and insulin levels, suppressing elevation of corticosterone and attenuating intestinal inflammation [127].

## Concluding comments

Several important conclusions can be drawn from this review:

1. P2Y_6_ receptors influence hypothalamic control of feeding, and the P2Y_6_ receptor antagonist MRS 2578 inhibits food intake in obese mice. Therefore, P2Y_6_ receptors are a potential therapeutic target for the prevention and treatment of obesity.
2. A_2A_ receptor agonists, acting on adipocytes, counteract HFD-induced obesity in mice, indicating A_2A_ receptors as a potential drug target for anti-obesity therapies.
3. P2Y_11_ receptors have stimulatory effects on lipolysis in adipocytes. Therefore, these receptors deserve further explorations.
4. Both P2X_7_ receptors, which mediate inflammation, and K_ATP_ are beginning to be explored for the treatment of obesity.
5. Growing lines of evidence suggest that a subtle balance of adipogenic and osteogenic differentiation of mesenchymal stem cells is crucial in tissue homeostasis and a loss of adipo-osteogenic balance leads to pathophysiological conditions, such as obesity.
6. P2Y_1_ receptors are responsible for the extracellular ATP-mediated intracellular triglyceride accumulation in adipocytes. The P2Y_1_ receptor antagonist MRS 2500 significantly inhibited triacylglycerol accumulation, suggesting the P2Y_1_ receptor as a novel therapeutic target for the treatment of lipid disorders.
7. P2Y_4_ receptors are negative regulators of cardiac adipose-derived stem cell differentiation and cardiac fat formation. Therefore, these receptors could be potential therapeutic targets in the regulation of the cardioprotective function of cardiac fat.
8. Activation of P2Y_13_ receptors mediates HFD- and palmitic acid-induced myenteric neuronal loss in mice. Myenteric neurons from mice lacking the P2Y_13_ receptors or treated with a selective P2Y_13_ receptor antagonist are resistant to HFD- and palmitic acid-induced loss. Antagonism of P2Y_13_ receptors might constitute a novel therapeutic strategy in patients with obesity affected by intestinal dysmotility.
9. Adipocyte ABC transporter G1 promoted triglyceride storage and fat mass growth. Thus, it might represent a potential therapeutic target in the control of fat accumulation.
10. A_1_ receptor agonists are in clinical trials for obesity. Over-expression of A_1_ receptors in adipose tissue protects mice from obesity-related insulin resistance.
11. A_2B_ receptors prevent HFD-induced hallmarks of type 2 diabetes, adipose tissue inflammation and insulin resistance. Therefore, the A_2B_ receptor might represent a possible therapeutic strategy.

Purinergic signalling offers proof-of-concept potential for the development of novel therapeutic approaches to treat obesity, mostly from studies in animal models. Currently, pharmacological obesity treatment options are palliative and limited. An excess of body fat is associated with cardiovascular disorders and metabolic syndromes, including insulin-independent diabetes and dyslipidemia; therefore, a combination of lifestyle changes and new drugs may be the most efficacious approach to achieving sustained weight loss for the majority of patients with obesity. In particular, strategies to combat obesity may include drugs that regulate bodyweight acting through CNS pathways or via peripheral adiposity signals and the gastrointestinal tract.

There are a number of promising studies on several animal models and systems that could be translated to human applications. Current data obtained with experimental models support the notion that the purinergic system consists of adenosine receptors, metabotropic P2Y receptors and ionotropic P2X_7_ receptors, which are all thought to contribute to the pathology of obesity. The enormous flexibility and diversity of the purinergic system can be exploited in drug design for therapeutic intervention and the development of anti-obesity drugs, although further understanding is needed. Indeed, the development of selective agonists and antagonists for the different purinergic receptor subtypes could be combined with the investigation of the interactions of purinergic signalling with other established signalling systems in relation to obesity. Hopefully, the potential use of purinergic compounds that are orally bioavailable and stable in vivo for the treatment of obesity will soon be prepared by medicinal chemists that can be used in clinical trials.
